# Supplementary material for: Patient perspectives of antiretroviral pharmacy services: A cross-sectional cohort study
Source: PLoS One. 2023 May 23;18(5):e0285694. doi: 10.1371/journal.pone.0285694 (PMC10204950; doi:10.1371/journal.pone.0285694)
Supplement: S1 Table — (PDF) [file pone.0285694.s001.pdf]

	Pharmacy Setting Preference[a]		
Characteristic	Local (n=41)	Mail-Order (n=16)	P-Value
Age, median (IQR)	53.0 (19)	47.0 (14.5)	0.189
Sex			
Male	37 (90.2)	16 (100.0)	0.568
Female	4 (9.8)	0 (0.0)	
Race			
White	34 (82.9)	14 (87.4)	0.184
Black	7 (17.1)	1 (6.3)	
Asian	0 (0.0)	1 (6.3)	
Ethnicity			
Hispanic	3 (7.3)	0 (0.0)	0.552
Not Hispanic	38 (92.7)	16 (100.0)	
Insurance Coverage			
Commercial	34 (82.9)	14 (87.4)	0.500
Medicare	5 (12.2)	1 (6.3)	
Medicaid	2 (4.9)	0 (0.0)	
VA/Government	0 (0.0)	1 (6.3)	
Treatment			
HIV Treatment	37 (90.2)	14 (87.4)	1.000
HIV Prevention (PrEP)	4 (9.8)	2 (12.6)	
Mail-Order Mandate Experience			
Mandate	35 (85.4)	11 (68.8)	0.260
No Mandate	6 (14.6)	5 (31.3)	
