# Supplementary material for: Patient perspectives of antiretroviral pharmacy services: A cross-sectional cohort study
Source: PLoS One. 2023 May 23;18(5):e0285694. doi: 10.1371/journal.pone.0285694 (PMC10204950; doi:10.1371/journal.pone.0285694)
Supplement: Attachment — Submitted filename: There are no comments.docx [file pone.0285694.s002.docx]

There are no comments, work is good can be published.
